# Aging and the intramyocardial inflammatory response

**DOI:** 10.1186/s13054-014-0638-2

**Published:** 2014-11-19

**Authors:** Keith R Walley

**Affiliations:** Centre for Heart Lung Innovation, St. Paul’s Hospital, University of British Columbia, 1081 Burrard Street, Vancouver, BC V6Z 1Y6 Canada

## Abstract

The sepsis-induced intramyocardial inflammatory response results in decreased ventricular function and myocardial damage. Chemokines such as monocyte chemoattractant protein-1 causally contribute to retention of intramyocardial mononuclear leukocytes and subsequent ventricular dysfunction during endotoxemic shock in mice and, importantly, this effect is age dependent. It is therefore useful to consider where monocyte chemoattractant protein-1 fits in the complex pathway leading to ventricular dysfunction during sepsis, why this might be an age-dependent effect, and what this implies for care of older sepsis patients.

Slimani and colleagues report that monocyte chemoattractant protein-1 (MCP-1) causally contributes to ventricular dysfunction during endotoxemic shock in mice and, importantly, this effect is age dependent [1]. It is therefore useful to consider where MCP-1 fits in the complex pathway leading to ventricular dysfunction during sepsis, why this might be an age-dependent effect, and what this implies for care of older sepsis patients.

Sepsis results in an intramyocardial inflammatory response that leads to decreased left ventricular contractility and diastolic dysfunction [2]. If the degree of ventricular dysfunction is minimal then the heart is able to increase cardiac output in response to sepsis-induced decreased systemic vascular resistance and increased venous return, which result in the familiar hyperdynamic septic circulation. Worsening septic shock is characterized by a progressive decrease in cardiac output despite aggressive volume resuscitation and decreased left ventricular afterload. In this circumstance – hypodynamic septic shock – it follows that ventricular dysfunction is a major causal contributor to adverse outcomes. Greater understanding of the intramyocardial inflammatory response is important when considering mitigating strategies.

Pathogen-associated molecular patterns, such as lipopolysaccharide (endotoxin) from Gram-negative organisms and peptidoglycan from Gram-positive organisms, bind innate immune receptors including Toll-like receptors (TLRs) [3]. TLRs are expressed on many cell types but particularly on cell lines involved in the earliest response to infecting pathogens, such as monocytes/macrophages. Subsequent intracellular signaling via nuclear factor-κB results in expression of an array of inflammatory cytokines [3].

Interestingly, cardiomyocytes express TLRs [4] (notably TLR2 and TLR4) and receptors for a variety of inflammatory cytokines so that circulating pathogen-associated molecular patterns and inflammatory cytokines [5] result in an intramyocardial inflammatory response. Cardiomyocytes respond with their own internal nuclear factor-κB signaling [4,6], production of cytokines such as interleukin-6, chemotactic cytokines (chemokines) such as MCP-1 [7] and keratinocyte chemoattractant (KC), production of nitric oxide [8], and upregulation of cell surface adhesion molecules such as intracellular adhesion molecule-1 [9]. Adhesion molecules are also expressed on activated coronary endothelial cells and contribute to retention of circulating leukocytes in the coronary circulation [10]. Leukocytes are then drawn into the myocardium [11] down chemokine gradients. Ligation of cardiomyocyte intracellular adhesion molecule-1 by leukocytes [9,12], fibrinogen [13], and other inflammatory molecules adversely impacts cardiomyocyte calcium handling, which results in decreased contractility [9]. This intramyocardial inflammatory response also triggers apoptotic pathways [14] that, even in the absence of significant end-stage cardiomyocyte apoptosis [15], can result in mitochondrial damage and dysfunction [16]. Thus, all aspects of the septic intramyocardial inflammatory response contribute to ventricular dysfunction. Whether chemokines such as MCP-1 and KC play a causal role or whether they are simply upregulated bystanders has not previously been fully elucidated.

A key observation by Slimani and colleagues is that increased myocardial MCP-1 expression resulted in increased numbers of intramyocardial mononuclear leukocytes and a greater decrease in ventricular contractility [1]. Fundamentally important is the further observation that this pathway causally contributes to decreased ventricular contractility because neutralization of MCP-1 abrogated these effects. The observation that similar neutralization of KC did not have the same effect provides an important negative control. These results suggest that MCP-1 attracts intramyocardial mononuclear leukocytes that then decrease contractility, as steps in a causal pathway. These investigators present left ventricular pressure and volume data so we can surmise that end-systolic elastance, as a load-independent measure of ventricular contractility, decreased with endotoxemia and was lowest in old mice.

Nuclear factor-κB signaling leading to cytokine and chemokine production increases with aging [17]. The consequences of this age-related exaggerated septic inflammatory response have not been fully explored, yet initial observations suggest that age-dependent increases in signaling via TLR4 induce greater ventricular dysfunction [18]. Slimani and colleagues have confirmed and extended these observations by considering cytokines and chemokines that have previously been found to be increased in the myocardium after a septic inflammatory stimulus (tumor necrosis factor alpha, interleukin-1β, interleukin-6, MCP-1, KC, macrophage inflammatory protein-1α), and they nicely demonstrate that increased MCP-1 does decrease contractility [1]. Neutralization of MCP-1 was approximately twice as effective in improving left ventricular ejection fraction in old mice compared with young adult mice. Age-dependent changes in inflammatory cytokine and chemokine production thus have important downstream functional effects.

These observations arise from a murine model so a number of limitations must be recognized. The peripheral blood leukocyte differential is quite different in mice, where most of the circulating leukocyte count is made up of mononuclear leukocytes. Neutrophils are under-represented compared with humans. Thus, the intramyocardial mononuclear infiltration, driven by the monocyte chemokine MCP-1, makes sense in this murine setting while lack of effect of KC, a granulocyte/neutrophil chemokine, also fits with the murine model. Nevertheless, the current results emphasize the functional importance of intramyocardial mononuclear leukocytes that are indeed observed in humans [19]. This murine model used endotoxin administration, which is not a realistic model of human sepsis but is an excellent tool to isolate and study specific pathways induced by pathogen-associated molecular patterns. We should thus be circumspect in extrapolating and interpreting the importance of this degree of ventricular dysfunction, but we can be confident in concluding that intramyocardial chemokines attracting leukocytes contribute to ventricular dysfunction and this response is exaggerated with aging.

In sum, these novel findings further support the notion that the inflammatory response and its cardiovascular consequences importantly change with age. Early intervention and resuscitation aimed at reducing the proinflammatory stimulus must continue to be emphasized in patients of all ages.

## Abbreviations

KC: Keratinocyte chemoattractant
MCP-1: Monocyte chemoattractant protein-1
TLR: Toll-like receptor
